# Conventional and new proposals of GnRH therapy for ovarian, breast, and prostatic cancers

**DOI:** 10.3389/fendo.2023.1143261

**Published:** 2023-03-28

**Authors:** Maritza P. Garrido, Andrea Hernandez, Margarita Vega, Eyleen Araya, Carmen Romero

**Affiliations:** ^1^ Laboratorio de Endocrinología y Biología de la Reproducción, Hospital Clínico Universidad de Chile, Santiago, Chile; ^2^ Departamento de Obstetricia y Ginecología, Facultad de Medicina, Universidad de Chile, Santiago, Chile; ^3^ Departamento de Ciencias Quimicas, Facultad de Ciencias Exactas, Universidad Andres Bello, Santiago, Chile

**Keywords:** GnRH, targeted therapy, ovarian cancer, breast cancer, prostatic cancer

## Abstract

For many years, luteinizing hormone-releasing hormone or gonadotropin-releasing hormone (GnRH) analogs have been used to treat androgen or estrogen-dependent tumors. However, emerging evidence shows that the GnRH receptor (GnRH-R) is overexpressed in several cancer cells, including ovarian, endometrial, and prostate cancer cells, suggesting that GnRH analogs could exert direct antitumoral actions in tumoral tissues that express GnRH-R. Another recent approach based on this knowledge was the use of GnRH peptides for developing specific targeted therapies, improving the delivery and accumulation of drugs in tumoral cells, and decreasing most side effects of current treatments. In this review, we discuss the conventional uses of GnRH analogs, together with the recent advances in GnRH-based drug delivery for ovarian, breast, and prostatic cancer cells.

## 1 Introduction

The luteinizing hormone-releasing hormone, herein called gonadotropin-releasing hormone (GnRH), is a peptide hormone synthesized and released in a pulsatile fashion by hypothalamic neurons. GnRH stimulates the synthesis and secretion of the gonadotropins follicle-stimulating hormone (FSH) and luteinizing hormone (LH) from the pituitary gland (1); therefore, it displays a critical role in reproductive physiology. Their synthesis is regulated by the feedback of circulating levels of gonadal hormones to maintain the homeostasis of reproductive function.

**Figure 1:**
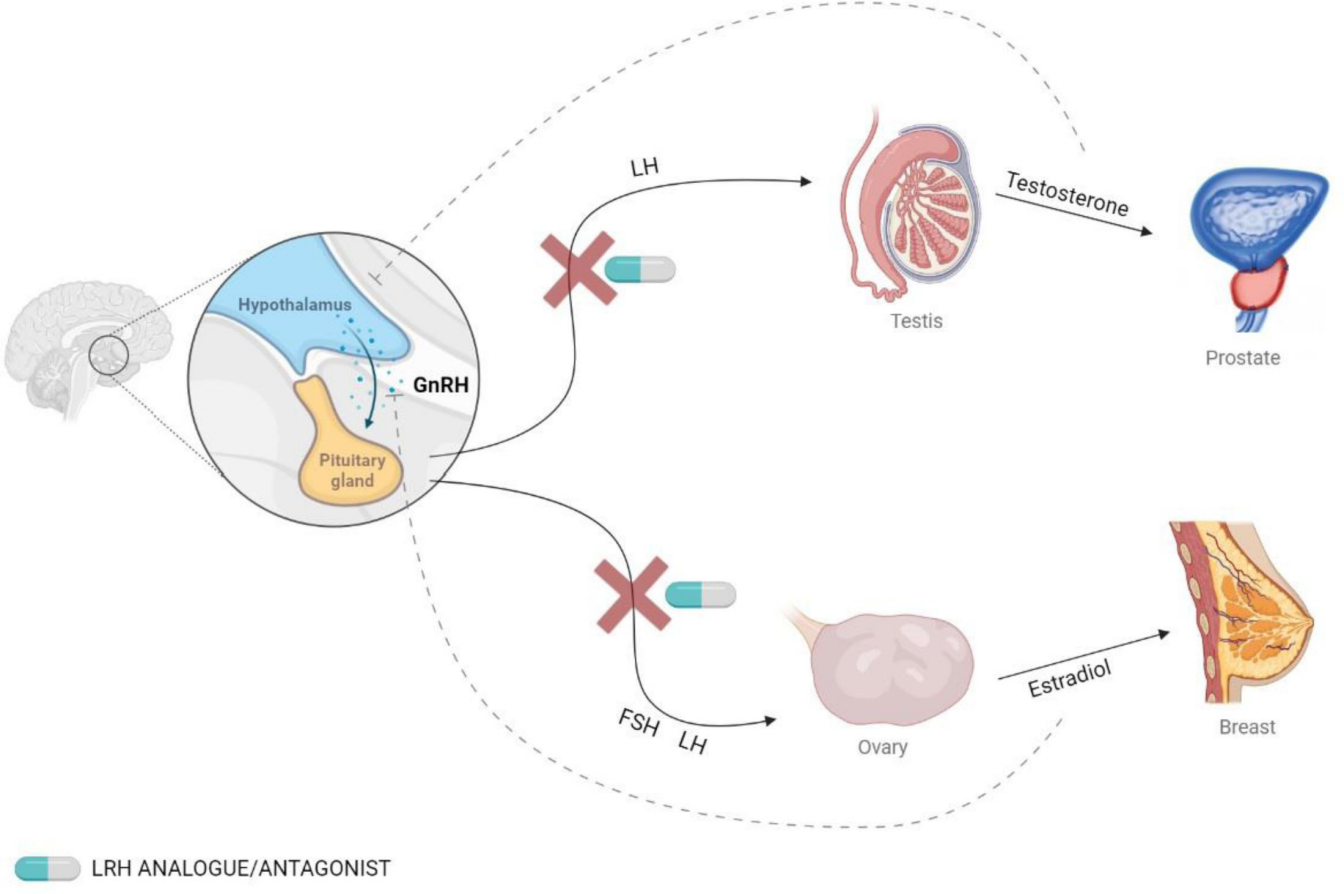
Secretion of GnRH and the effects on hormonal levels. GnRH is secreted by the hypothalamus and binds to its receptor in the pituitary gland, which stimulates the release of follicle-stimulating hormone (FSH) and luteinizing hormone (LH). This effect can be blocked using GnRH analogs.

The use of antagonists or long-acting analogs of GnRH disrupts de endocrine axis and decreases the endocrine function of gonads, producing medical castration. This treatment is widely used in some neoplasms that express androgen or estrogen receptors and whose growth is encouraged by circulating gonadal hormones (2, 3). For instance, in the case of breast cancer tissue, the immunodetection of estrogen receptors (ER) and progesterone receptors (PR) in biopsies is currently included as part of the clinical routine. Abundant evidence relates the expression of these receptors with patients’ prognosis and the response to endocrine therapy (4). In a similar manner, prostatic tissue expresses androgen receptors, and 30-50% of prostatic cancers show amplification of the androgen receptor gene, producing its overexpression (5). This knowledge has promoted the use of GnRH agonists and antagonists to induce castration in patients with breast (6) and prostatic cancer (7) (**Figure 1**).

GnRH agonists produce an increase in the secretion of gonadotropin hormones, but continuous use leads to a downregulation of the receptors, which ultimately causes a decrease in gonadal hormone levels. On the other hand, GnRH antagonists produce an immediate inhibition of gonadotropin secretion (8). The most commonly used GnRH agonists are leuprolide, triptorelin, and goserelin, and the most common antagonists are degarelix and relugolix (9, 10).

**Figure 2:**
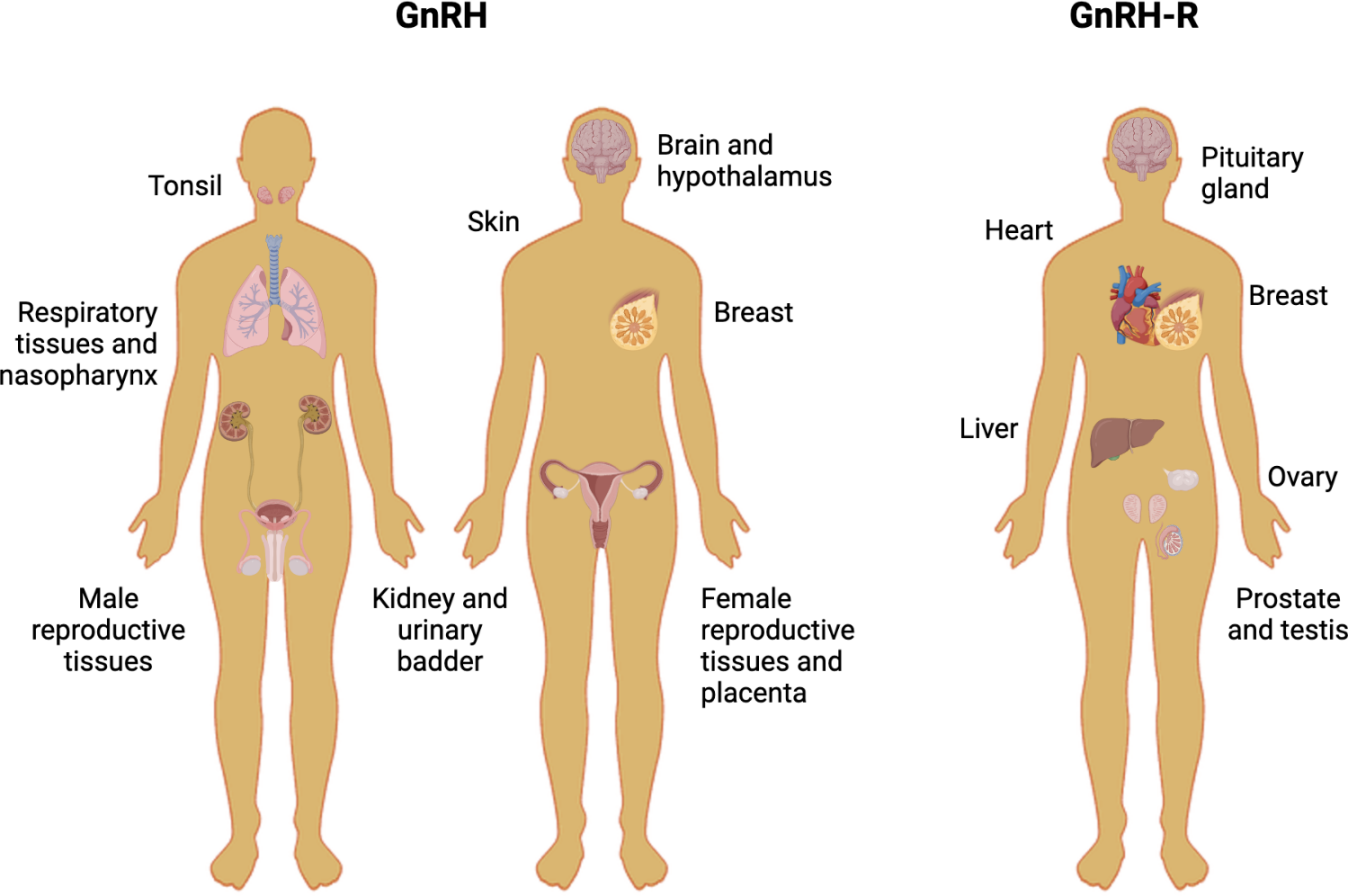
Human tissues that express GnRH and GnRH-R. The picture shows different human non-tumoral tissues in which the protein levels of GnRH and GnRH-R have been assessed.

Moreover, GnRH could exert direct effects in non-pituitary tissues, involving the activation of GnRH receptors (GnRH-R) (see **Figure 2**). The expression of GnRH and/or GnRH-R has been reported in the liver, heart, skeletal muscle, kidney, breast, and reproductive tissues, as well as malignant tumors of the breast, gonads, and urogenital tract (11–21). We will review diverse experiments using cell lines from breast, ovary, endometrium, and prostatic cancers, which have shown that GnRH analogs produce anti-tumoral effects, mainly reducing cell proliferation, tumoral size, and metastasis *in vitro* and *in vivo.*

Besides the direct and indirect anti-tumoral effects of GnRH, another potential therapeutic approach is their use in targeting therapy. As indicated below, GnRH-drug conjugates or nanoparticle complexes conjugated with GnRH and chemotherapeutical agents have been developed to treat tumors that express GnRH-R. These formulations allow selective delivery and exhibit several advantages, such as the improvement of drug internalization and accumulation of chemotherapeutics in cancer cells, thus minimizing side effects.

## 2 Use of GnRH analogs in androgen deprivation therapy

Prostate cancer is the most common cancer in men and the second one in respect of mortality (22). Risk factors include advanced age, ethnicity, and family history. Most cases are detected when the cancer is localized, and the 5-year survival rate is around 83% (23). The treatment for localized prostate cancer includes surgery and radiation, while metastatic prostate cancer is treated with chemotherapy and androgen deprivation therapy (ADT) (24).

The growth of almost 70% of prostate cancer cases is testosterone-dependent (25). Hence, GnRH agonists have been used since 1980 to treat prostatic cancer (26, 27) and currently, they are the first line of treatment. In advanced stages of the disease, combined androgen blockade (CAB) is recommended; this includes the use of chemical castration plus an anti-androgen. The use of CAB in the early stages of cancer is still under debate (10, 28, 29). GnRH agonists could be administered subcutaneously daily, even though there are slow-release formulations, such as microcapsules and implants that can release GnRH analogs for up to three months (25).

In its initial stage, prostatic cancer is largely dependent on androgens; however, some patients can develop over time an androgen-independent cancer. Antecedents have shown that FSH promotes the progression of this androgen-independent cancer, being capable of stimulating proliferation and decreasing apoptosis in an *in vitro* model (30, 31). Importantly, FSH levels are reduced ten times with the use of GnRH antagonists (32), in contrast with patients that undergo surgery and whose levels of FSH remain high. When cancer becomes androgen-independent, there is no consensus on whether to continue the GnRH therapy in these patients, and the decision is made considering the patient’s quality of life (33). The withdrawal of the GnRH therapy in some of these patients might contribute to the progression of androgen-independent prostate cancer.

To delay the progression of prostatic cancer to an androgen-independent phenotype, a new protocol of intermittent androgen suppression has been proposed. It is based on the adaptative mechanism of survival of the cancer cells in an environment without androgens, which can be postponed by giving the patient time off therapy to recover normal testosterone levels. The results of this protocol were not conclusive regarding the patient’s survival or cancer progression, but there is evidence that this protocol enhances their quality of life, with fewer side effects and a better sexual life, along with economic benefits (34, 35).

The adverse effects of GnRH therapy usually are impotence, osteoporosis, and dyslipidemia (36). ADT has also been associated with an increased risk of diabetes (37). These side effects should be treated preventively to improve the life quality of the patients (38).

## 3 GnRH analogs for breast and ovarian cancer

Breast cancer is the most common cancer in women, especially in middle-aged and older women, and the leading cause of mortality due to cancer in this group (22). The risk factors include: age, gender, family history, use of hormonal contraceptives, and hormonal replacement therapy (39). Around 62% of the cases are diagnosed in the early stages, in which approximately 90% of the cases have a 5-year survival rate (40, 41). Breast cancer therapy consists of surgery, radiation, chemotherapy, and endocrine-based therapy, which includes GnRH analogs (42). 70% of women and over 80% of men with breast cancer are estrogen receptor-positive, and they are treated with tamoxifen, which blocks the estrogen receptor from binding to its ligand (43–45). Tamoxifen is used by both premenopausal and postmenopausal women, but postmenopausal women are mostly treated with aromatase inhibitors, which decrease the levels of estrogens by inhibiting the aromatase that produces estrogen (46, 47).

Around 80% of breast cancer in women and over 80% of breast cancer in men are hormone receptor-positive (48–50). As is known, estrogen and progesterone exert an important role in the progression of this cancer due to the effect of sex hormones on the proliferation of cancer cells (51). GnRH therapy is used as an adjuvant treatment in breast cancer to prevent recurrence and prolong patient survival (6). In rare cases, some patients have contraindications for tamoxifen, in which case GnRH therapy is used together with an aromatase inhibitor (42). GnRH by itself has been successfully used in premenopausal women, with a response in up to 63% of the patients, but only in 22% of postmenopausal women (52). In addition, tamoxifen and GnRH therapy can improve the overall survival of patients with breast cancer (53).

In the case of breast cancer cells, the interaction of FSH and LH with their receptors produces changes in the expression of genes related to adhesion, motility, and invasion (54). Therefore, GnRH therapy could be relevant to decrease FSH and LH levels in postmenopausal women, who have higher levels of gonadotropins. In this context, GnRH treatment would be considered adequate in the case of triple-negative breast cancer (TNBC), because half of these tumors upregulate GnRH-R (55). TNBC is estrogen-receptor (ER) negative, progesterone-receptor (PR) negative, and HER2 negative (56).

Among the subtypes of breast carcinoma, TNBC has a poor prognosis and shows the worst clinical outcomes. Unfortunately, due to a lack of molecular targets, the treatment for TNBC requires new therapeutic alternatives. A systematic revision of 4 investigations performed by Corona et al. (57) showed that the use of GnRH analogs could increase the overall survival of TNBC patients, in comparison to the control arm, although the difference was not statistically significant. However, most of the trials evaluated in the analysis were designed to test the efficacy of GnRH analogs to prevent premature ovarian failure in premenopausal women during adjuvant chemotherapy, which may represent a limitation. The study suggests that GnRH analogs could be useful as a targeted therapy in TNBC; therefore, clinical trials are needed to evaluate this alternative.

On the other hand, GnRH analogs could be an interesting alternative for the treatment of other cancer types, such as ovarian cancer. Despite its low incidence, ovarian cancer is the second cause of death due to gynecological cancer and over two-thirds of the cases are diagnosed in women of ages 55 or older (22). Among the risk factors are: hormonal replacement therapy, uninterrupted ovulation cycles, and family history (58). The treatment consists of cytoreductive surgery and chemotherapy (59) and this neoplasm is usually diagnosed in the late stages, when the 5-year survival rate is around 47% among all ages (60, 61).

Most ovarian cancer tissues express GnRH-R, as well as the receptors for FSH, LH, and estradiol. The interaction of these ligands and their receptors increases cell proliferation of ovarian cells (62–65). In contrast, the use of GnRH analogs decreases the proliferation of ovarian cancer cells *in vitro (* 13).

Currently, GnRH analogs are not clinically used to treat ovarian cancer. Their use has been evaluated in several clinical trials, showing modest efficacy (66). In patients with platinum-resistant ovarian cancer, the GnRH analog, Leuprolide, and the antagonist Cetrorelix have been tested. While 9% and 18% of patients had partial remission, 26% and 35% of patients showed disease stabilization, respectively (67, 68). *In vivo* studies in ovarian carcinoma resistant to platinum chemotherapy showed that the use of both GnRH analogs and chemotherapy produces cytotoxic effects in ovarian cancer xenografts, with a significant reduction in the volume of ovarian tumors (14, 69).

In the long term, GnRH therapy can produce symptoms of menopause, fertility impairment, blood pressure changes, osteoporosis, and increase the risk of coronary heart disease (6, 70). However, in general, GnRH analogs have less severe side effects than chemotherapy and can be more specific in targeting hormone-dependent cancers. However, there is a need to improve the delivery methods to target a specific organ and thus, minimize the adverse effects.

## 4 Use of GnRH-based compounds for cancer therapy

Several studies have been performed to improve current anti-tumoral therapies, but with modest significant advances in cancer treatment. Most drugs used in conventional therapeutic strategies have low solubility, high metabolism, and are hydrophobic, and these features make these drugs biologically unavailable and can lead to systemic toxicity (71). Furthermore, standard chemotherapeutic treatments are limited in their selectivity toward tumor sites, and produce multiple drug resistance in tumoral cells (71). Another common problem related to cancer chemotherapy is drug toxicity and side effects, since they are designed to rapidly destroy dividing cells, including those found in healthy tissues (72).To overcome these problems, the development of drug-targeted therapies could increase drug efficacy and decrease the side effects of anticancer drugs (73).

In this context, carrier-based drug delivery systems (polymer conjugates, liposomes, micelles, dendrimers, nanogels, inorganic or other solid particles, and others) are being widely investigated to overcome the limitations of conventional drug chemotherapy and improve its overall safety and patient convenience (74). Numerous preclinical and clinical studies employing delivery systems have shown a better therapeutic effect and reduced overall toxicity, attributed mainly to a controlled drug release profile (75). **Table 1** summarizes the studies that have tested nanoformulations in breast, prostate, and ovarian cancer.

**Table 1:** Summary of different nanosystems that use GnRH analogs to increase the selective delivery of drugs to *in vivo* and *in vitro* models of ovarian, prostatic, and breast cancer.

Target	Nanosistem	Main findings	Reference
Prostatic cancer	[D-Lys6]-GnRH with methotrexate	*In vitro*: ↓ Cell proliferation ↑ Cell cytotoxicity of methotrexate ↑ Apoptosis *In vivo*: ↓ tumor volume and tumoral weight	(76)
Prostatic cancer	GnRH-conjugated micelles loaded with an antiandrogen	*In vitro*: ↓ cell viability ↑ cellular uptake of the antiandrogen drug ↑ induction of apoptosis *In vivo*: ↓ Tumor volume ↑ Tumoral apoptosis markers	(77)
Ovarian cancer	Gold nanoparticles carrying GnRH and doxorubicin	*In vitro*: ↑ Cell death ↑ doxorubicin accumulation	(78)
Prostatic cancer	Goserelin (GnRH analog)-conjugated gold nanorods	*In vitro*: ↑ Goserelin uptake ↓ Clonogenic survival fraction *In vivo*: ↑Tumor-growth delays	(79)
Ovarian cancer	GnRH-nanogels with ionic cores loaded with cisplatin	*In vivo*: ↓ Tumor volume ↑ Survival rate of animals	(80)
Breast cancer	GnRH-ferrosoferric oxide	*In vitro*: ↑cell cytotoxicity *In vivo*: ↑ Selective accumulation in the tumor	(81)
Breast cancer	GnRH-targeted cisplatin-loaded dextran nanoparticles	*In vitro*: ↑ platinum uptake and cell cytotoxicity *In vivo*: ↑ platinum accumulation ↓ Tumor volume	(82)
Breast cancer	Human serum albumin-methotrexate functionalized with GnRH	*In vitro*: ↑ cell cytotoxicity (↓ IC50) *In vivo*: ↑ survival rate of animals	(83, 84)
Breast cancer	Mitoxantrone-loaded liposomas with GnRH	*In vitro*: Equal cytotoxicity of free drug by low release rate	(85)

Arrows indicate an increase (↑) or decrease (↓) of tumoral processes.

**Figure 3:**
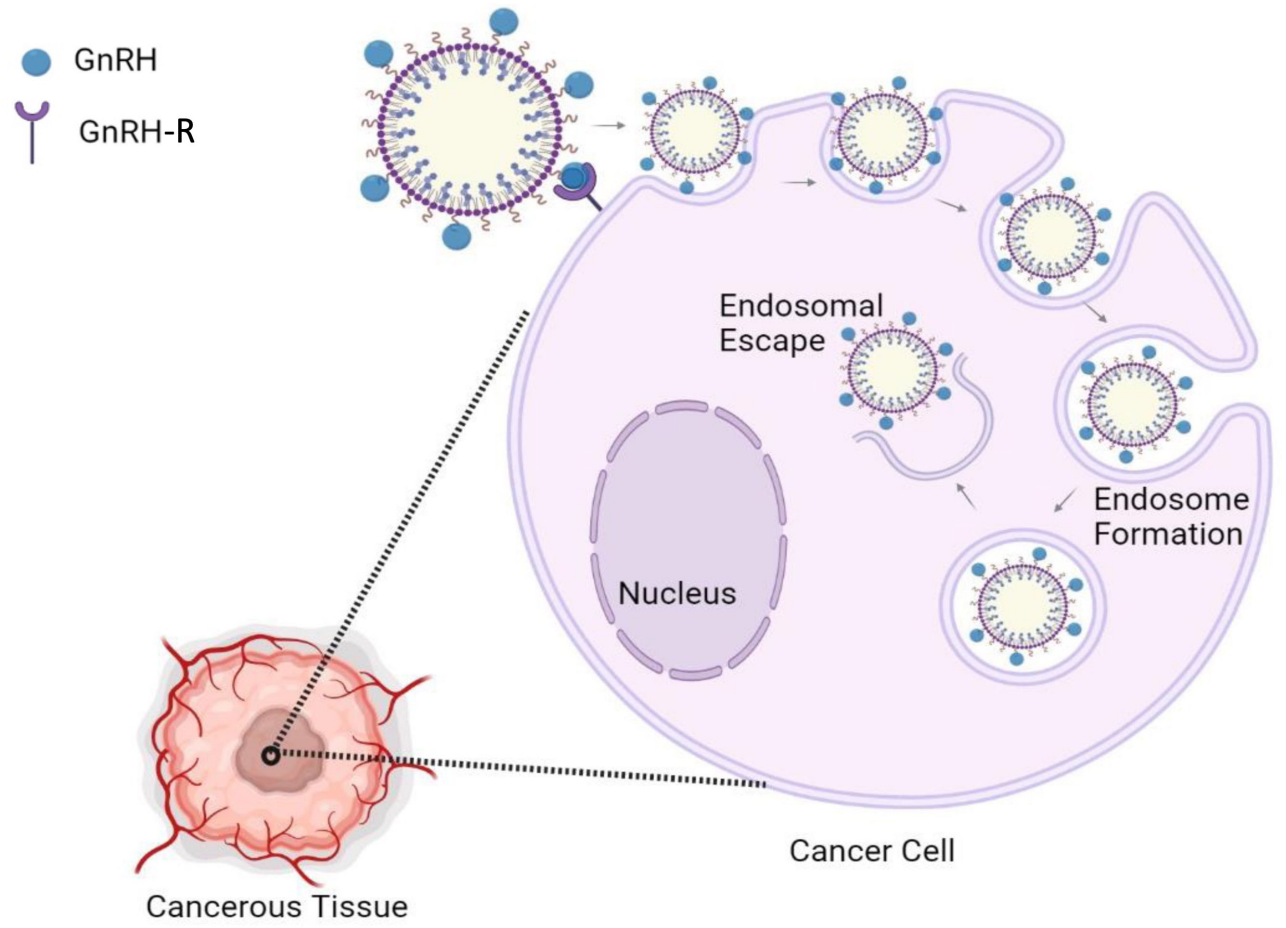
Input mechanism of GnRH-based nanoparticles for cancer cells. Nanoparticles coated with GnRH interact with the GnRH-R, promoting the entrance into cancer cells *via* endosome formation. Then, the nanoparticle content is released inside the cell.

Receptors that are primarily expressed in cancer cells represent attractive molecular targets for selective drug delivery. The binding of GnRH to GnRH-R appears to lead to receptor microaggregation and internalization of the peptide (86) (see **Figure 3**). The GnRH-R is overexpressed in most cancers, but its expression in healthy tissues, excluding pituitary cells, is limited (87–89). Accordingly, recent studies have indicated that GnRH peptides could be used as an efficient guide of anticancer compounds and imaging agents, which can selectively target tumor cells, increase the amount of these substances in tumor tissue, and prevent normal cells from unnecessary exposure. Active targeting of cancer cells is a strategy based on the modification of anticancer agents and/or drug-loaded nanoparticles with targeting ligands that specifically bind to the receptors preferentially expressed or highly overexpressed in cancer cells (90–93).

Some studies have evaluated the use of GnRH peptide agonist and antagonist analogs in cancer tissues expressing GnRH-R (12–14). These studies employed GnRH-R-targeted dendrimers (94), nanoparticles (83), and liposomes (95), among others, to substantially increase the intra-tumor accumulation of anti-tumoral substances and therefore, enhance their anticancer efficacy. Importantly, GnRH-R-mediated targeting is independent of the nano-carrier architecture, composition, size, and molecular mass (95). Some of these studies used the [D-Lys6]-GnRH analog because it is resistant to degradation and is selectively accumulated in the nucleus of human GnRH receptor-positive breast, ovarian, and endometrial cancer cell lines (83, 96).

Nano-formulations have also been used for the detection of tumors and/or metastases through imaging techniques and to evaluate the treatment of different cancer types that have GnRH receptors. For instance, some studies employed GnRH peptides to achieve targeted delivery of radio-nucleotides as imaging agents (which attach to GnRH using chelating compounds) for their use in positron emission tomography (PET) and single photon emission computed tomography (SPECT) (95, 97). A GnRH conjugate that demonstrated rapid accumulation in both breast and prostate tumors, and specific binding to the GnRH receptor was developed and provided an efficient visualization of cancer lesions by SPECT (95, 98).

Studies suggest that GnRH peptides could act as local regulators of tumor growth (62, 99, 100). GnRH-R overexpression has been detected in hormone-dependent cancer tissues, such as breast (101), endometrial (102), ovarian (88, 103, 104), and prostate cancer (105); and also in hormone-independent tissues, such as pancreatic cancer (106, 107), lung cancer (108), melanoma (109), and glioblastoma (110). Moreover, GnRH-R expression levels are increased in various tumoral tissues. For example, GnRH-R is expressed in about 86% of prostate cancer, 80% of human endometrial and ovarian cancers, 80% of renal cancer, 50% of breast cancer, and 32–50% of pancreatic cancer cases (89, 111–114).

## 5 GnRH-based nano-formulations in prostatic and ovarian cancer

Currently, androgen ablation is a commonly prescribed treatment for localized prostatic cancer. However, this treatment has a limited scope, especially for hormone-refractory prostate cancers (115). Since prostate cancer tumor cells express the GnRH-R, some nano-formulations systems have been developed to deliver chemotherapy agents, producing less toxicity, and limiting nonspecific activity.

An interesting study performed in *in vivo* and *in vitro* prostatic cancer models tested the use of [D-Lys6]-GnRH with methotrexate ([D-Lys6]-GnRH-MTX). These results showed that prostatic cell growth was inhibited more by [D-Lys6]-GnRH-MTX than MTX alone, and that [D-Lys6]-GnRH-MTX also decreased tumor volume (74% vs 62% of MTX alone) and tumoral weight (74% vs 63% respectively) (76). On the other hand, Wen and coworkers (77) reported that GnRH-conjugated micelles loaded with the antiandrogen C4-2 cells exhibited a higher cellular uptake, promoting increased cell cytotoxicity and apoptosis, and efficient inhibition of prostatic cancer cell proliferation *in vitro* (approximately 80% of inhibition in C4-2 cells) and tumor growth *in vivo* (33% compared to only CBDIV17 micelles) after treatment (77).

On the other hand, peptides such as GnRH have been widely used for targeting nanoparticles to tumor cells in gynecological cancers, such as ovarian cancer. Due to the high expression of GnRH-R in ovarian cancer (compared with normal ovaries), nanoparticles containing GnRH can interact with its receptor, which leads to an endocytic process that facilitates cell internalization. The use of nanoparticles with GnRH has been developed with a focus on drug delivery and therapy in cancer treatment. Moreover, gold nanoparticles (GNPs) can be functionalized with molecules to achieve selective delivery to tumor cells, including a GnRH analog (78). Additionally, a potent radiosensitization of prostate cancers *in vitro* and *in vivo* using goserelin-conjugated gold nanorods has been reported (79). In this context, the study shows that treatment with goserelin-conjugated gold nanorods plus radiotherapy delayed tumor regrowth of a mouse xenograft by 17 ± 1 days compared to radiotherapy alone.

In the context of ovarian cancer diagnosis, gold nanoformulations could be used not only as a selective drug delivery agent but also as a diagnostic tool for imaging technologies, promoting non-invasive and real-time monitoring. For instance, a recent work tested GnRH-conjugated gold nanoparticles in a mouse model of ovarian cancer to assess their use in multi-energy spectral photon-counting computed tomography (116). The authors evidenced a preferential uptake of GnRH-gold nanoparticles in organs of the abdominal cavity, suggesting that this technology has potential use for imaging of ovarian cancer.

Another type of nanoformulation, a GnRH-cisplatin nanogel, was designed by Nukolova et al (80) to increase cell specificity and drug accumulation in ovarian cancer cell lines. This study showed that the cisplatin accumulation was specific for the GnRH-receptor-positive cells, more effective, and less toxic than equimolar doses of free cisplatin. Therefore, GnRH-targeted cisplatin enhanced the anti-tumoral effect of this drug in an animal model of ovarian cancer, decreasing the size of the tumor xenografts (approximately 40% less volume at day 25, compared to nanogels with only cisplatin) and increasing the survival rate of the animals (60% vs 15% respectively) (80).

Some GnRH analogs have been tested in patients; for instance, a conjugate of doxorubicin-GnRH agonist (AEZS-108) was tested in a phase 2 study performed in patients with metastatic hormone-resistant prostate cancer (NCT01240629) and demonstrated clinical benefit in 56% of the patients (progression-free survival at 12 weeks with no dose-limiting toxicities that require treatment cessation) (117). The same drug was tested in patients with chemotherapy-refractory triple negative breast cancer (NCT01698281), but the clinical trial was terminated due to poor recruitment.

## 6 GnRH-based nano-formulations in breast cancer

As mentioned before, the incidence of breast cancer has increased over the years, and even though there are various therapeutic strategies, the mortality rate has not decreased, particularly in TNBC (22). GnRH-R is a possible target of TNBC cells, and its expression and receptor kinetics have been well characterized (118, 119). These studies showed that the binding of GnRH to GnRH-R is increased in TNBC cells, indicating the existence of interactions between the overexpressed GnRH receptors and their ligands.

Both thermodynamics and kinetic models, and also *in vitro* experiments showed that GnRH conjugated with polyethylene glycol (PEG)-coated magnetite nanoparticles (GnRH-MNPs) can interact with TNBC and non-tumoral breast cells. This study suggested that GnRH-MNPs preferentially enter into TNBC cells *via* the receptor-mediated endocytosis pathway, with a significant GnRH-MNP uptake after 3 h (119). The same group also determined that the entrance of GnRH-MNPs to TBNC cells depends on its high efficiency to bind to the GnRH-R (119), suggesting that GnRH-MNPs can be used for the specific targeting of TNBC cells for both cancer detection and treatment. Similar conclusions were found by Nian et al (81), who synthetized GnRH-ferrosoferric oxide (GnRH-Fe_3_O_4_) nanoparticles. This formulation showed higher concentrations in the tumor (under the effect of a magnetic field *in vivo*, and most importantly, without evidence of heart, liver, or lung toxicity (81). This study concluded that GnRH-Fe_3_O_4_ nanoparticles could be useful for targeting contrast agents or targeted imaging, and for the treatment of cancers with high GnRH-R expression, such as breast cancer.

Another type of nanoformulation was designed by Li et al. (82), who tested GnRH-targeted cisplatin-loaded dextran nanoparticles in a model of metastatic breast cancer. These GnRH-based nanoparticles significantly increased the accumulation of cisplatin in the primary and metastatic tumors (twice as much as with cisplatin alone), reduced drug delivery to kidneys, and improved its anticancer activity; decreasing tumor volume by 49% compared to free cisplatin (82).

On the other hand, GnRH has been used to functionalize endogenous proteins, such as human serum albumin (HSA). For instance, HAS-methotrexate conjugates were functionalized with GnRH to achieve better incorporation into breast cancer cells, producing a significant rise in methotrexate internalization and its antitumoral activity in GnRH-R-positive breast cancer cells (IC50 of 49.2 vs 5.8 nM for non-targeted nanoparticles and GnRH-targeted nanoparticle respectively) (84). *In vivo* results of this formulation show a 2-fold increase in the percentage of animal survival compared to MTX alone (83). This strategy was carried out to improve the delivery of hydrophobic drugs such as MTX, promoting its reuse after dismissing its use due to its high rate of side effects.

Another therapeutic approach that has been considered for the treatment of breast cancer is the development of GnRH-targeted liposomes and micelles. He and coworkers (85) studied the delivery of mitoxantrone using GnRH analogs modified with PEGylated (polyethylene glycol thioether bond) liposomes. *In vitro* studies in MCF-7 cells (a metastatic adenocarcinoma cell line) with high expression of GnRH-R revealed that targeted liposomes showed higher internalization and sustained drug release characteristics. However, the release rate of the drug was low, which depressed the action of mitoxantrone on tumor cells, suggesting that it is necessary to continue improving this type of formulations (85).

## 7 Main conclusions

GnRH-R is overexpressed in many types of cancer and therefore, many drug delivery strategies developed for cancer-specific therapy have been used for GnRH targeting. Recent efforts are aimed at designing and developing new drug delivery systems using GnRH peptide/analogs as targeting moieties. Diverse studies also revealed positive results with many types of GnRH- nanoformulations in terms of binding, accumulation, and treatment efficacy.

Numerous studies have reported the use of the GnRH peptide or its analogs as a targeting ligand to increase the potency of chemotherapeutic drugs, demonstrating the efficiency of the specific binding between GnRH peptide/analog-based carriers and the GnRH-R in cancer cells/tumors. *In vitro* and *in vivo* experiments have reported enhanced internalization of the drugs into cancer cells and accumulation in the tumor site, confirming the effectiveness of the new GnRH-targeted delivery. One of the most attractive strategies could be the use of GnRH-targeted nanoparticles, which have shown an increase in selective drug accumulation and promising results in models of ovarian, breast, and prostatic cancer. However, up to now, only two trials have tested GnRH-targeting therapy and therefore, its clinical use is still in its early initial stages.

## Author contributions

Writing – Original Draft Preparation: MG, AH, and CR. Writing – Review & Editing: MG, MV, EA, and CR. All authors contributed to the article and approved the submitted version.
